# Long-term survival after intensive chemotherapy or hypomethylating agents in AML patients aged 70 years and older: a large patient data set study from European registries

**DOI:** 10.1038/s41375-021-01425-9

**Published:** 2021-11-13

**Authors:** Christian Récher, Christoph Röllig, Emilie Bérard, Sarah Bertoli, Pierre-Yves Dumas, Suzanne Tavitian, Michael Kramer, Hubert Serve, Martin Bornhäuser, Uwe Platzbecker, Carsten Müller-Tidow, Claudia D. Baldus, David Martínez-Cuadrón, Josefina Serrano, Pilar Martínez-Sánchez, Eduardo Rodríguez Arbolí, Cristina Gil, Juan Bergua, Teresa Bernal, Adolfo de la Fuente Burguera, Eric Delabesse, Audrey Bidet, Arnaud Pigneux, Pau Montesinos

**Affiliations:** 1 Centre Hospitalier Universitaire de Toulouse, Institut Universitaire du Cancer de Toulouse Oncopole, Université Toulouse III Paul Sabatier, Toulouse, France; 2 Medizinische Klinik und Poliklinik I, Universitätsklinikum TU Dresden, Dresden, Germany; 3 Centre Hospitalier Universitaire de Toulouse, Service d’Epidémiologie, CERPOP, Inserm, Université Toulouse III Paul Sabatier, Toulouse, France; 4 CHU Bordeaux, Service d’Hématologie Clinique et de Thérapie Cellulaire, Université de Bordeaux, Institut National de la Santé et de la Recherche Médicale, U1035, 33000 Bordeaux, France; 5 Medizinische Klinik 2, Universitätsklinikum Frankfurt, Frankfurt/Main, Germany; 6 Klinik und Poliklinik für Hämatologie, Zelltherapie und Hämostaseologie, Universitätsklinikum Leipzig, Leipzig, Germany; 7 Klinik für Hämatologie, Onkologie und Rheumatologie, Universitätsklinikum Heidelberg, Heidelberg, Germany; 8 Klinik für Innere Medizin II, Universitätsklinikum Schleswig-Holstein, Kiel, Germany; 9 Instituto de Investigación Sanitaria La Fe (IISLAFE), Hospital Universitari i Politècnic La Fe, Valencia, Spain; 10 Hospital Universitario Reina Sofía-IMIBIC, Córdoba, Spain; 11 Hospital Universitario 12 de Octubre, Madrid, Spain; 12 Hospital Universitario Virgen del Rocío, Sevilla, Spain; 13 Hospital General Universitario de Alicante, Alicante, Spain; 14 Hospital San Pedro Alcántara, Cáceres, Spain; 15 Hospital Universitario Central de Asturias, Asturias, Spain; 16 MD Anderson Cancer Center Madrid, Madrid, Spain; 17 Centre Hospitalier Universitaire de Toulouse, Institut Universitaire du Cancer de Toulouse Oncopole, Laboratoire d’Hématologie Biologique, Toulouse, France; 18 CHU Bordeaux, Laboratoire d’Hématologie Biologique, F-33000 Bordeaux, France

**Keywords:** Acute myeloid leukaemia, Chemotherapy

## Abstract

The outcome of acute myeloid leukemia patients aged 70 years or older is poor. Defining the best treatment option remains controversial especially when choosing between intensive chemotherapy and hypomethylating agents. We set up a multicentric European database collecting data of 3 700 newly diagnosed acute myeloid leukemia patients ≥70 years. The primary objective was to compare overall survival in patients selected for intensive chemotherapy (*n* = 1199) or hypomethylating agents (*n* = 1073). With a median follow-up of 49.5 months, the median overall survival was 10.9 (95% CI: 9.7–11.6) and 9.2 months (95% CI: 8.3–10.2) with chemotherapy and hypomethylating agents, respectively. Complete remission or complete remission with incomplete hematologic recovery was 56.1% and 19.7% with chemotherapy and hypomethylating agents, respectively (*P* < 0.0001). Treatment effect on overall survival was time-dependent. The Royston and Parmar model showed that patients treated with hypomethylating agents had a significantly lower risk of death before 1.5 months of follow-up; no significant difference between 1.5 and 4.0 months, whereas patients treated with intensive chemotherapy had a significantly better overall survival from four months after start of therapy. This study shows that intensive chemotherapy remains a valuable option associated with a better long-term survival in older AML patients.

## Introduction

The prognosis of acute myeloid leukemia (AML) patients aged ≥70 years is particularly poor. In the United States, five-year overall survival is 7.4% and 3.3% in patients aged 70–74 and 75–79, respectively [1]. Similar results have been reported in Europe [2]. Indeed, these patients accumulate adverse risk factors related both to aging and to distinct disease characteristics [3]. Older patients more often have co-morbidities and/or a more compromised performance status at diagnosis [4, 5]. In addition, they more often present disease-related characteristics associated with a poorer prognosis such as complex karyotype, *ASXL1*, *RUNX1* or *TP53* mutations and/or secondary AML evolving from myelodysplastic syndrome or myeloproliferative disorders or after previous exposure to cytotoxic treatments (therapy-related AML) [6, 7]. This results in poor treatment tolerance, higher treatment-related mortality and more failure or relapse than in younger subjects and explains why recent improvement in overall survival observed in AML is less pronounced in older patients [1, 8, 9].

In elderly patients, intensive chemotherapy (IC), hypomethylating agents (HMAs), low-dose cytarabine (LDAC) or best supportive care (BSC) represent standard treatment options. More recently, the addition of the Bcl2 inhibitor venetoclax to azacitidine or low-dose cytarabine has shown efficacy by improving response to treatment and overall survival in patients judged ineligible for intensive chemotherapy [10, 11].

Accurately determining which kind of treatment is most appropriate for which patient remains a daily challenge, particularly in selecting older patients who are suitable for intensive treatment, which is the only therapeutic option associated with long term survival [12]. Scoring systems have been proposed to rationalize clinical decision-making [4, 13–19]. However, many patients and physicians are reluctant to use induction chemotherapy due to its toxicity and disappointing results, especially in patients over 75 years of age [20]. In fact, age ≥75 years has become a criterion for non-eligibility to IC in recent clinical trials and for novel drug indications. Over the past decade, HMAs have emerged as an alternative to IC in this specific situation [21]. Although these therapeutic strategies have not been formally compared in a prospective randomized trial, most retrospective studies have shown similar median overall survival rates [22–24]. A recent single-center study even reported a survival benefit in favor of HMAs [25]. Thus, there is a dilemma between two options, one associated with greater toxicity but higher response rate and a possibility of long-term survival, and the other, better tolerated, producing fewer responses and no real hope of survival beyond three years. Apart from this, IC requires only short term therapy while HMA treatment should be continued lifelong thus also interfering with long-term quality of life.

In this study, we thought to gather a sufficient number of older European AML patients to compare the effect of IC and HMA treatments by multivariate and propensity score matched analyses. We therefore collected demographic and therapeutic data from 3 700 patients aged 70 years or older with the aim of comparing patients routinely selected for IC or HMAs.

## Subjects and methods

### Patients

Individual patient data were collected from three European AML registries: Toulouse–Bordeaux DATAML, Study Alliance Leukemia (SAL) and Programa Español de Tratamientos en Hematología (PETHEMA). All patients 70 years of age or older with AML newly diagnosed between January 1, 2007, and June 30, 2018 were included. Acute promyelocytic leukemia cases were not included. A minimal data set was collected for each patient, including the variables age, sex, date of diagnosis, AML status (de novo or secondary), white blood cell count, percentage of peripheral and bone marrow blasts, LDH, cytogenetic risk, *NPM1*, *FLT3*-ITD, *CEBPA*, *IDH1*, *IDH2* mutational status, nature of first-line therapy, response to treatment, allogeneic hematopoietic stem cell transplantation in first complete remission, date of relapse and/or death. This study was performed in accordance with the Declaration of Helsinki. All registries were approved by institutional review boards or national authorities, and informed consent was obtained from all patients.

### Treatments and endpoints

First-line treatments included intensive induction chemotherapy combining an anthracycline (idarubicin or daunorubicin) and cytarabine with or without a third drug such as lomustine, a semi-intensive regimen, HMAs (azacitidine or decitabine), low-dose cytarabine (LDAC) or best supportive care. Chemotherapy regimens routinely used by the three study groups have been published elsewhere [9, 26–28]. Semi-intensive regimens in the PETHEMA group included fludarabine, cytarabine, and filgrastim [27]. Allogeneic hematopoietic stem cell transplantation could be offered in selected patients mainly after intensive chemotherapy. Bone marrow assessment in patients treated with intensive chemotherapy was performed after blood recovery or, in case of delayed recovery between days 35 and 45. In the HMA group, bone marrow aspiration was carried out after 3 to 6 cycles. Response to treatment, relapse, relapse-free survival (RFS) and overall survival (OS) were defined according to the European Leukemia Net (ELN) criteria [29].

### Statistical analysis

Statistical analyses were performed using STATA statistical software, version 16.1 (STATA Corp., College Station, TX). We described the patients’ characteristics using numbers and frequencies for qualitative data, and medians with inter-quartile ranges (IQR) for quantitative data. Comparisons between the patients’ characteristics were assessed using Student’s *t*-test (or the Mann–Whitney test when the distribution departed from normality or when homoscedasticity was rejected) for continuous variables, and the χ2-test (or Fisher’s exact test when there were small expected numbers) for categorical variables. Then, OS and RFS for HMAs vs. IC were described using Kaplan-Meier curves. Because the proportional hazards assumption was not respected for the effect of HMAs vs. IC, we used a Royston and Parmar survival model [30]. Differences in early death and response rate were compared between treatments using a logistic regression model. Multivariate analyses included HMAs vs. IC together with potential confounding factors [age ≥75 y, performance status >1, white blood cell count at diagnosis >30 giga per liter, cytogenetic risk, secondary vs de novo AML, *NPM1* mutation, *FLT3*-ITD mutation (for RFS) and study period]. Stepwise analysis was then used to assess variables that were significantly and independently associated with the endpoints. Interactions between all potential confounding factors and treatment (HMAs vs. IC) were tested. None were significant, indicating that the effect of HMAs vs. IC is not significantly different according to all confounding factors analyzed (and in particular according to age, performance status, cytogenetic risk or *NPM1* mutation). To better appreciate the impact of HMAs vs. IC, we used the propensity score method to more extensively take into account potential baseline differences between HMAs and IC subjects. A multivariate logistic regression model was generated to estimate for each patient a propensity score to receive HMAs vs. IC. Covariates were all variables expected to be associated with HMAs vs. IC in clinical practice (age, performance status, WBC, cytogenetic risk, secondary vs de novo AML, *NPM1* and *FLT3*-ITD mutations, study period and center). The performance of the model was estimated with the χ2-Hosmer–Lemeshow statistic and the C-statistic. Based on propensity score, subjects with IC were matched with subjects with HMAs and endpoints were compared between HMAs and IC in the subgroup of propensity score matched subjects. All reported p-values were two-sided and the significance threshold was <0.05.

## Results

### Study population

**Fig. 1:**
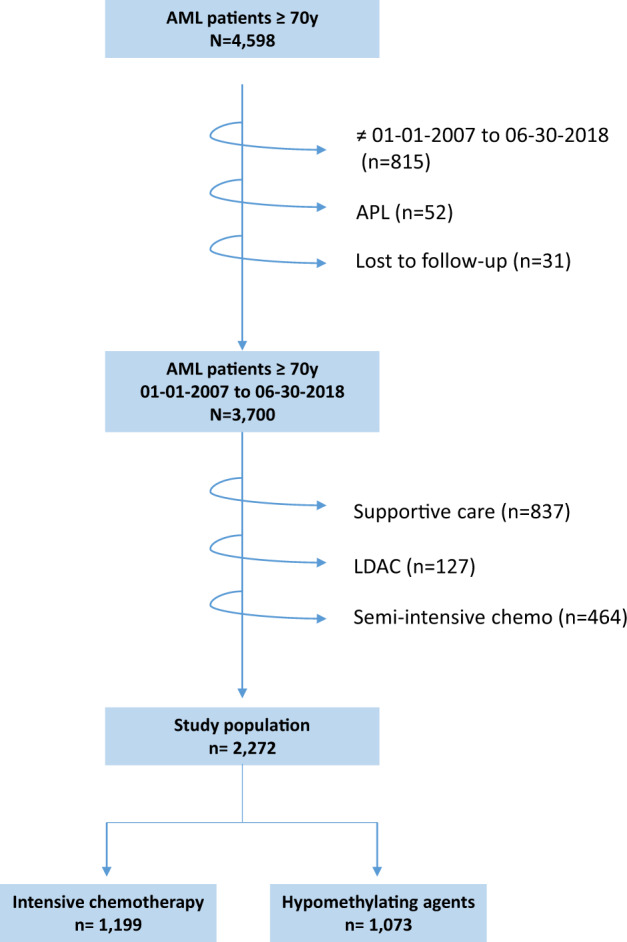
Study flowchart. APL acute promyelocytic leukemia. LDA low-dose cytarabine.

A total of 3700 AML patients ≥70 years of age with sufficient data were identified (Fig. 1). Patients treated with semi-intensive chemotherapy (*n* = 464), LDAC (*n* = 127) or supportive care (*n* = 837) were not included in the primary analysis (see characteristics and outcome in Supplementary Table 1 and 2). Thus, the study population for the principal objective of this study included 1 199 patients treated with IC and 1 073 patients treated with HMAs. Their characteristics are presented in Table 1. The median follow-up was 49.5 months (interquartile range, 29.1–75.0). HMAs were more frequently used in the recent period (2013–2018). This trend was not different between patients aged 70–74 years and patients ≥75 years. In the HMA group, patients were older, had lower WBC count and bone marrow blast percentages, and they more frequently had ECOG performance status >1, secondary AML and adverse-risk cytogenetics as compared to the IC group. *NPM1* and *FLT3*-ITD mutations were more frequent in the IC group. The main IC regimens were daunorubicin-cytarabine (*n* = 432, 36.0%), idarubicin–cytarabine (*n* = 381, 31.8%) or idarubicin-cytarabine-lomustine (*n* = 214, 17.8%) combinations. Inclusion in a clinical trial involved 141 patients (11.8%) treated with IC and 210 (19.6%) treated with HMA (*P* < 0.001) without the addition of venetoclax or other antineoplastic agents. Allogeneic hematopoietic stem cell transplantation was performed in 70 patients (5.8%) treated with IC and only in seven patients (0.7%) treated with HMAs (*P* < 0.001).

**Table 1:** Characteristics of the 2272 AML patients ≥70 years according to treatment.

	Intensive chemotherapy *N* = 1199 (52.8%)	Hypomethylating agents *N* = 1073 (47.2%)	*P* value	All patients *N* = 2272 (100%)
Study period – no. (%)				
2007–2012	532 (44.4)	346 (32.2)	< 0.001	878 (38.6)
2013–2018	667 (55.6)	727 (67.8)		1394 (61.4)
Sex – no. (%)				
Male	669 (55.8)	611 (57.0)	0.563	1280 (56.4)
Female	529 (44.2)	460 (43.0)		989 (43.6)
Age – years				
Median (IQR)	74.0 (72.0–76.0)	77.5 (74.0–81.1)	<0.001	75.0 (72.5–79.0)
<75 y – no. (%)	740 (61.7)	331 (30.8)		1071 (47.1)
≥75 y – no. (%)	459 (38.3)	742 (69.2)		1201 (52.9)
ECOG performance status – no. (%)				
0–1	846 (74.9)	651 (68.2)	<0.001	1497 (71.8)
2–4	283 (25.1)	304 (31.8)		587 (28.2)
AML status – no. (%)				
De novo	848 (75.3)	572 (58.7)	<0.001	1420 (67.6)
Secondary	278 (24.7)	402 (41.3)		680 (32.4)
White blood cell count – giga per liter				
Median (IQR)	9.3 (2.3–54.0)	3.5 (1.8–12.3)	<0.001	5.2 (2.0–27.3)
≤30 – no. (%)	784 (66.4)	923 (88.3)		1707 (76.7)
>30 – no. (%)	396 (33.6)	122 (11.7)		518 (23.3)
Peripheral blasts – %				
Median (IQR)	30.0 (6.0–71.0)	10.0 (2.0–33.0)	<0.001	19.0 (3.0–56.0)
Bone marrow blasts - %				
Median (IQR)	61.0 (38.0–82.0)	36.0 (25.0–60.0)	<0.001	50.0 (30.0–75.0)
LDH – IU/liter				
Median (IQR)	429.0 (258.0–736.0)	396.3 (239.4–600.0)	<0.001	396.0 (248.0–676.0)
Cytogenetic risk – no. (%)				
Favorable	47 (4.4)	9 (1.0)	<0.001	56 (2.9)
Intermediate	795 (75.2)	518 (59.6)		1313 (68.2)
Adverse	215 (20.3)	342 (39.4)		557 (28.9)
*NPM1* mutations				
No	553 (64.4)	390 (81.2)	<0.001	943 (70.4)
Yes	306 (35.6)	90 (18.8)		396 (29.6)
*FLT3*-*ITD* mutations – no. (%)				
No	698 (80.2)	463 (91.0)	<0.001	1161 (84.2)
Yes	172 (19.8)	46 (9.0)	0.232	218 (15.8)
Allelic ratio – no	134	40		174
Median (IQR)	0.6 (0.3–0.8)	0.4 (0.2–0.9)		0.6 (0.3–0.9)
*IDH1*-R132 mutations – no. (%)				
No	187 (90.8)	239 (89.5)	0.649	426 (90.1)
Yes	19 (9.2)	28 (10.5)		47 (9.9)
*IDH2-R140* mutations – no. (%)				
No	178 (86.4)	234 (88.3)	0.538	412 (87.5)
Yes	28 (13.6)	31 (11.7)		59 (12.5)
*IDH2-R172* mutations – no. (%)				
No	204 (98.6)	257 (97.7)	0.737	461 (98.1)
Yes	3 (1.4)	6 (2.3)		9 (1.9)
Inclusion in a clinical trial – no. (%)				
No	1058 (88.2)	863 (80.4)	<0.001	1921 (84.6)
Yes	141 (11.8)	210 (19.6)		351 (15.4)
Allogeneic stem cell transplantation – no. (%)				
No	1129 (94.2)	1066 (99.3)	<0.001	2195 (96.6)
Yes	70 (5.8)	7 (0.7)		77 (3.4)

### Response to treatment

Complete remission or complete remission with incomplete hematologic recovery (CR/CRi) was achieved in 673 (56.1%) and 211 (19.7%) patients in the IC and HMA groups, respectively (*P* < 0.0001). Multivariate analysis of factors associated with CR/CRi is shown in Table 2. Age ≥ 75 years, ECOG performance status >1, adverse-risk cytogenetics and WBC count >30 giga/liter were significantly and independently associated with a lower response rate whereas *NPM1* mutation was significantly and independently associated with a higher response rate. After adjusting for these factors, the choice of first-line treatment was also significantly and independently associated with response, meaning that HMA treatment was associated with a lower response rate than IC (odds-ratio (OR), 0.25; 95% CI: 0.20–0.31; *P* < 0.001).

**Table 2:** Multivariate analysis for response to treatment and early mortality.

	Number	Events	aOR	95% CI	*P* value
	CR/CRi				
Treatment					
Intensive chemotherapy	1199	673	1		
Hypomethylating agents	1073	211	0.25	0.20–0.31	<0.001
Age - years					
<75	1071	525	1		
≥75	1201	359	0.69	0.57–0.84	<0.001
ECOG performance status					
0–1	1497	661	1		
2–4	587	159	0.52	0.41–0.65	<0.001
AML status					
De novo	1420	617	1		
Secondary	680	213	0.81	0.65–1.01	0.064
Cytogenetic risk					
Favorable	56	37	1		
Intermediate	1313	610	0.54	0.30–0.98	0.043
Adverse	557	148	0.32	0.17–0.59	<0.001
White blood cell count – giga per liter					
≤30	1707	644	1	0.52–0.84	
>30	518	222	0.66		0.001
*NPM1* mutations					
No	943	382	1	1.36–2.36	
Yes	396	230	1.79		<0.001

	Day-30 death				
Treatment					
Intensive chemotherapy	1199	156	1		
Hypomethylating agents	1073	93	0.61	0.45–0.82	0.001
ECOG performance status					
0–1	1497	122	1		
2–4	587	104	2.26	1.69–3.02	<0.001
Cytogenetic risk					
Favorable	56	3	1		
Intermediate	1313	123	1.96	0.60–6.46	0.268
Adverse	557	68	3.26	0.97–10.93	0.055
White blood cell count – giga per liter					
≤30	1707	151	1		
>30	518	95	1.99	1.47–2.69	<0.001

	Day-60 death				
Treatment					
Intensive chemotherapy	1199	247	1		
Hypomethylating agents	1073	194	0.69	0.54–0.88	0.003
Age - years					
<75	1071	187	1		
≥75	1201	254	1.29	1.02–1.61	0.030
ECOG performance status					
0–1	1497	222	1		
2–4	587	187	2.50	1.99–3.16	<0.001
Cytogenetic risk					
Favorable	56	5	1		
Intermediate	1313	207	1.97	0.77–5.06	0.160
Adverse	557	143	4.12	1.59–10.73	0.004
White blood cell count – giga per liter					
≤30	1707	300	1		
>30	518	136	1.56	1.21–2.02	0.001

*aOR* adjusted odds ratio, *CI* confidence interval, *CR* complete remission, *CRi* complete remission with incomplete hematologic recovery.

Interaction between treatment (hypomethylating agents vs. intensive chemotherapy) and age (< vs ≥75 y), performance status (≤ vs >1), cytogenetic risk (favorable vs. intermediate vs. adverse) or *NPM1* mutation (yes vs. no) was not significant, showing that the effect of hypomethylating agents vs. intensive chemotherapy was not significantly different according to age, performance status, cytogenetic risk and *NPM1* mutation. Thus, there is no indication to stratify the analysis of age, performance status, cytogenetic risk and *NPM1* mutation (the OR for hypomethylating agents vs. intensive chemotherapy shown in Table 2 was the same according to age (< vs ≥75 y), performance status (≤ vs >1), cytogenetic risk (favorable vs. intermediate vs. adverse) or NPM1 mutation (yes vs. no)).

### Early mortality

Early mortality was evaluated at day 30 and day 60 of treatment in both groups. Day-30 death occurred in 156 (13.0%) and 93 (8.7%) patients in the IC and HMA groups, respectively (*P* = 0.001). Multivariate analysis of factors associated with day-30 death showed that ECOG performance status >1 and WBC count >30 giga/liter were significantly and independently associated with a higher day-30 death rate (Table 2). After adjustment for these factors, the choice of first-line treatment was also significantly and independently associated with day-30 death meaning that HMA treatment was associated with a lower day-30 death rate than IC (OR, 0.61; 95% CI: 0.45–0.82; *P* = 0.001).

Day-60 death occurred in 247 (20.6%) and 194 (18.1%) patients in the IC and HMA groups, respectively (*P* = 0.129). Multivariate analysis of factors associated with day-60 death showed that age ≥ 75 years, ECOG performance status >1, adverse-risk cytogenetics and WBC count >30 giga/liter were significantly and independently associated with a higher day-60 death rate (Table 2). After adjusting for these factors, the choice of first-line treatment was also significantly and independently associated with day-60 death, meaning that HMA treatment was associated with a lower day-60 death rate than IC (OR, 0.69; 95% CI: 0.54–0.88; *P* = 0.003).

### Overall and relapse-free survival

**Fig. 2:**
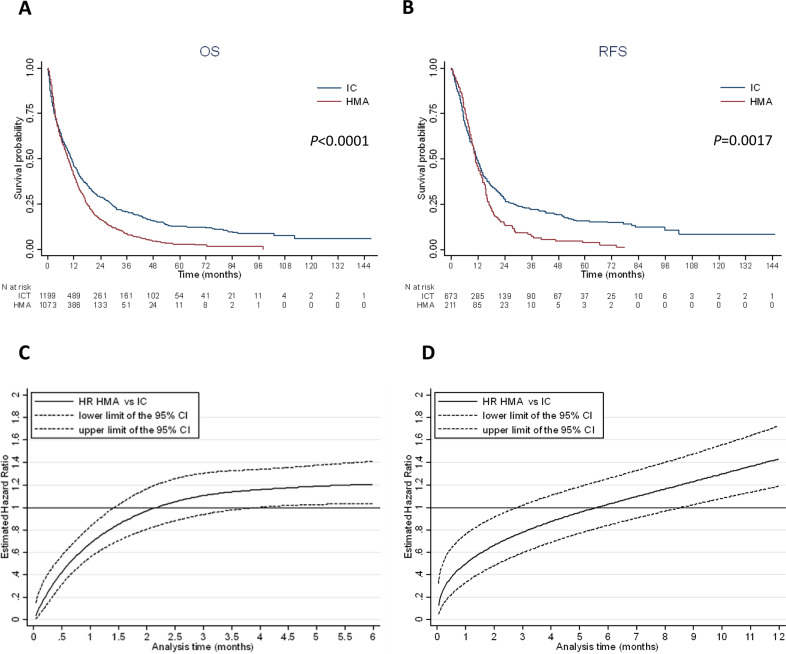
Survival according to intensive chemotherapy or HMA treatment. **A** Kaplan–Meier curve of overall survival according to treatment (median OS: 10.9 months, 95% CI: 9.7–11.6 with IC and 9.2 months, 95% CI: 8.3–10.2 with HMAs). **B** Kaplan–Meier curve of relapse free survival according to treatment (median RFS: 11.5 months, 95% CI: 10.5–12.7 with IC and 11.0 months, 95% CI: 9.7–12.9 with HMAs). **C** Royston and Parmar adjusted* hazard ratio for overall survival in HMA vs. IC for each month from diagnosis. Before 1.5 months of follow-up, patients treated with HMAs had a significantly lower risk of death compared to IC patients. Between 1.5 months and 4.0 months of follow-up, there was no significant difference in survival between HMAs and IC patients. From 4.0 months of follow-up, patients treated with HMAs had a significantly higher risk of death compared to IC patients. Interaction between treatment (HMAs vs. IC) and age (< vs ≥75 y), performance status (≤ vs >1), cytogenetic risk (favorable vs. intermediate vs. adverse) or *NPM1* mutation (yes vs. no) was not significant, showing that the effect of HMAs vs. IC was not significantly different according to age, performance status, cytogenetic risk and *NPM1* mutation. Thus, there is no indication to stratify the analysis on age, performance status, cytogenetic risk and *NPM1* mutation (Figure C was the same according to age (< vs. ≥75 y), performance status (≤ vs. >1), cytogenetic risk (favorable vs. intermediate vs. adverse) or *NPM1* mutation (yes vs. no)). *Adjusted for age ≥75 y, performance status > 1, white blood cell count at diagnosis >30 giga per liter, cytogenetic risk, secondary vs de novo AML and *NPM1* mutation. **D** Royston and Parmar adjusted* hazard ratio for relapse-free survival in HMAs vs. IC for each month from CR/CRi. Before 3 months of follow-up, patients treated with HMAs had a significantly lower risk of relapse or death compared to IC patients. Between 3 months and 8.5 months from CR/CRi, there was no significant difference between HMAs and IC patients. Beyond 8.5 months from CR/CRi, patients treated with HMA had a significantly higher risk of relapse or death compared to IC patients. Interaction between treatment (HMAs vs. IC) and age (< vs. ≥75 y), performance status (≤ vs. >1), cytogenetic risk (favorable/intermediate vs. adverse) or *NPM1* mutation (yes vs. no) was not significant, showing that the effect of HMAs vs. IC was not significantly different according to age, performance status, cytogenetic risk and *NPM1* mutation. Thus, there is no indication to stratify the analysis on age, performance status, cytogenetic risk and *NPM1* mutation (Figure D was the same according to age (< vs. ≥75 y), performance status (≤ vs. > 1), cytogenetic risk (favorable/intermediate vs. adverse) or *NPM1* mutation (yes vs. no)). *Adjusted for performance status >1, white blood cell count at diagnosis >30 giga per liter, cytogenetic risk, secondary vs. de novo AML, *NPM1* and *FLT3*-ITD mutations.

The median overall survival was 10.9 (95% CI: 9.7–11.6) and 9.2 months (95% CI: 8.3–10.2) in the IC and HMA groups, respectively. Overall survival at one, three and five years was 46.0 (95% CI: 43.0–48.9) vs. 40.6% (95% CI: 37.6–43.7), 20.8 (95% CI: 18.3–23.4) vs. 8.3% (95% CI: 6.5–10.4) and 12.4 (95% CI: 10.2–14.9) vs 2.8% (95% CI: 1.7–4.4) in the IC and HMA groups, respectively (Fig. 2A). In multivariate analysis, ECOG performance status >1, adverse-risk cytogenetics, WBC count >30 giga/liter and secondary AML were significantly and independently associated with a poorer overall survival (Table 3). Furthermore, long-term survival (>3 years) was associated with a higher achievement of CR with IC (Supplementary Table 3).

**Table 3:** Multivariate analysis for overall and relapse free survival.

	Number	Events	aHR	95% CI	*P* value
	Overall survival				
Treatment					
Intensive chemotherapy	1199	928			
Hypomethylating agents	1073	914	See R&P (Fig. 2C)^a^		
Age - years					
<75	1071	839	1		
≥75	1201	1003	1.19	1.08–1.31	<0.001
ECOG performance status					
0–1	1497	1189	1		
2–4	587	507	1.56	1.41–1.74	<0.001
AML status					
De novo	1420	1108	1		
Secondary	680	597	1.21	1.09–1.34	<0.001
Cytogenetic risk					
Favorable	56	41	1		
Intermediate	1313	1023	0.91	0.67–1.25	0.570
Adverse	557	495	1.73	1.25–2.39	0.001
White blood cell count – giga per liter					
≤30	1707	1396	1		
>30	518	413	1.40	1.25–1.57	<0.001
*NPM1* mutations					
No	1383	1146	1		
Yes	546	409	0.82	0.71–0.95	0.010

	Relapse-free survival				
Treatment					
Intensive chemotherapy	673	497			
Hypomethylating agents	211	179	See R&P (Fig. 2D)^a^		
ECOG performance status					
0–1	661	500	1		
2–4	159	131	1.24	1.02–1.52	0.031
AML status					
De novo	617	461	1		
Secondary	213	174	1.30	1.08–1.56	0.004
Cytogenetic risk					
Favorable/ Intermediate	647	484	1		
Adverse	148	126	1.58	1.28–1.95	<0.001
White blood cell count – giga per liter					
≤30	644	500	1		
>30	222	167	1.19	0.98–1.45	0.077
*FLT3*-*ITD* mutations					
No	511	386	1		
Yes	114	90	1.33	1.03–1.73	0.029
*NPM1* mutations					
No	382	303	1		
Yes	230	160	0.71	0.57–0.87	0.001

aHR, adjusted hazard ratio; CI, confidence interval.

^a^R&P: see Royston & Parmar.

Among the 673 CR/CRi patients in the IC group, 405 (60.2%) relapsed, whereas 139 out of 211 CR/CRi patients in the HMA group (65.9%) relapsed. Median relapse-free survival was 11.5 months (95% CI: 10.5–12.7) and 11.0 months (95% CI: 9.7–12.9) in the IC and HMA groups, respectively (Fig. 2B). It should be noted that relapse-free survival estimates may be biased in favor of IC given the much later time point when remission status was first assessed in patients treated with HMAs (median time between the beginning of treatment and the date of CR: 3.0 months [IQR:1.9–5.6] in the HMA group vs 1.3 months [IQR:1.1–1.8] in the IC group; p < 0.0001). In multivariate analysis, *NPM1* mutation was significantly and independently associated with a better relapse-free survival whereas ECOG performance status >1, adverse-risk cytogenetics, *FLT3*-ITD mutation and secondary AML were associated with a poorer relapse-free survival (Table 2).

Of note, the study period (2007–2012 vs. 2013–2018) was not independently associated with CR/CRi, early death rates, relapse-free and overall survival.

### Time-dependent treatment effect

The treatment effect on relapse-free and overall survival was time-dependent. To account for the non-proportionality of risks, we used a Royston and Parmar model, which took into account the interactions between time and treatment effect and allowed graphical representation of the adjusted risk of death (or of the adjusted risk of relapse or death for relapse-free survival) at all times during follow-up (Fig. 2C, D). This model showed that patients treated with HMAs had a significantly lower risk of death than patients treated with IC before 1.5 months of follow-up; there was no significant difference between the HMA and IC groups between 1.5 months and 4.0 months, and overall survival was significantly better in the IC group from 4.0 months of follow-up (Fig. 2C). Similarly, for relapse-free survival, patients treated with HMAs had a significantly lower risk of relapse or death than patients treated with IC before 3 months of follow-up; there was no significant difference between 3.0 months and 8.5 months, and relapse-free survival was significantly better in the IC group from 8.5 months of follow-up (Fig. 2D).

Of note, interactions between treatment (HMAs vs. IC) and all confounding factors were tested (for relapse-free survival, overall survival, CR/CRi and early death models). None were significant, indicating that the effect of HMAs vs. IC is not significantly different according to confounding factors (and in particular according to age, performance status, cytogenetic risk or *NPM1* mutation). Thus, there is no indication to stratify the analysis in these subgroups. Nevertheless, for information, we showed the results according to age (< vs ≥ 75 y) in Supplementary Table 4 and Supplementary Fig. 1.

### Propensity score matching

**Fig. 3:**
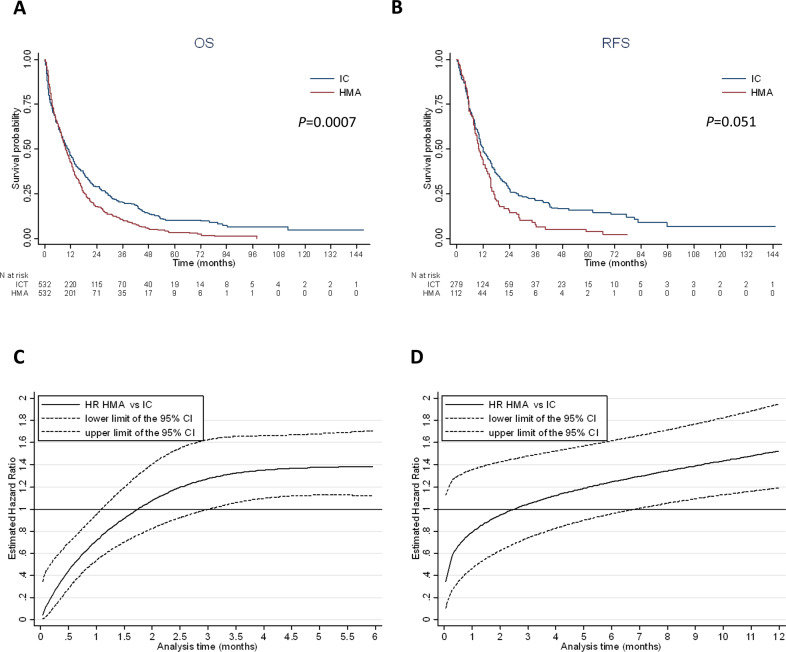
Survival according to intensive chemotherapy or HMA treatment in the pairwise population matched by the propensity score. **A** Kaplan–Meier curve of overall survival according to treatment in 532 IC patients matched with 532 HMA patients (median OS: 10.5 months, 95% CI: 8.8–12.2, with IC and 9.6 months, 95% CI: 8.5–11.0, with HMAs). **B** Kaplan–Meier curve of relapse free survival according to treatment (median RFS: 11.9 months, 95% CI: 10.3–14.5, with IC and 10.0 months, 95% CI: 8.4–12.9, with HMAs). **C** Royston and Parmar hazard ratio for overall survival in HMAs vs. IC for each month from diagnosis. Before one month of follow-up, patients treated with HMAs had a significantly lower risk of death compared to IC patients. Between 1 month and 3.0 months of follow-up, there was no significant difference in survival between HMA and IC patients. From 3.0 months of follow-up, patients treated with HMAs had a significantly higher risk of death compared to IC patients. **D** Royston and Parmar hazard ratio for relapse-free survival in HMAs vs. IC for each month from CR/CRi. Before 7 months of follow-up from CR/CRi, there was no significant difference in relapse or death between HMA and IC patients. From seven months of follow-up after CR/CRi, patients treated with HMAs had a significantly higher risk of relapse or death compared to IC patients.

To better estimate the impact of treatment on endpoints, we used the propensity score method to more extensively take into account potential baseline differences between HMA and IC subjects. A multivariate logistic regression model was generated to estimate for each patient a propensity score to receive HMAs or IC. The performance of the model was estimated with the χ^2^-Hosmer–Lemeshow statistic (*P* value = 0.169) and the C-statistic (0.82, 95% CI: 0.81–0.84). The mean propensity score was 0.320 (±0.232) in IC (*N* = 1199) and 0.642 (±0.234) in HMA (*N* = 1073). Based on propensity score, 532 subjects with IC were matched with 532 subjects with HMAs (630 with a precision of 0.0001, 18 with a precision of 0.001, 148 with a precision of 0.01 and the last with a precision of 0.1). The mean propensity score was the same in IC and HMA (0.491 ± 0.219) in the matched sample. The results of HMAs vs. IC comparisons on response, early mortality, disease-free survival and overall survival in this subgroup of propensity score-matched patients were similar to those of the multivariate analysis (Supplementary Table 5 and Fig. 3). Median relapse-free survival was 11.9 (95% CI: 10.3–14.5) and 10.0 (95% CI: 8.4–12.9) months in the IC and HMA groups, respectively. Median overall survival was 10.5 (95% CI: 8.8–12.2) and 9.6 (95% CI: 8.5–11.0) months in the IC and HMA groups, respectively. Again, the treatment effect on relapse-free and overall survival was time-dependent. The Royston and Parmar model assessing the evolution of hazard ratios according to time is presented in Fig. 3.

## Discussion

This study is the largest multicenter comparison of the two most commonly used front-line therapies in AML patients ≥70 years. With a fairly long median follow-up in this patient population, we showed that IC remains the treatment strategy that offers better chances for prolonged survival compared with HMAs. No significant interaction was found between treatments and independent variables indicating that the effect of treatment was not significantly different across the different subsets of patients, including those aged ≥75 years, with a poor performance status or even with an adverse cytogenetic risk.

After a decade of experience with HMAs in older AML patients, efforts to make comparisons with IC have been challenging and controversial, both prospectively and retrospectively [21]. In the prospective AZA-AML-001 randomized trial, IC was part of a conventional care regimen together with LDAC and best supportive care making a head-to-head comparison virtually impossible. Indeed, in this trial comparing azacitidine vs. a conventional care regimen, only 44 patients were assigned to IC [31]. Retrospective studies, which were most often monocentric and underpowered, yielded contradictory results [22–25]. A recent single-center study from the Moffitt Cancer Center conducted in AML patients ≥70 years showed opposite results to our analysis [25]. In this study, HMA treatment was associated with a significant better overall survival compared to IC. However, there are several important differences between both studies regarding the baseline characteristics of patients that may explain these discrepancies. These differences concerned the rates of secondary AML (56.9% vs. 32.4% in our study) and performance status > 1 (19% vs. 28%), white blood cell count (median, 3.3 vs. 5.2 giga per liter) and *NPM1* mutations (12.2% vs. 29.6%). Thus, the patient population of the Moffit Cancer Center presented more often with AML with myelodysplastic syndrome-like features, suggesting a center-related recruitment bias compared to our multicentric cohort. Study periods were also different with a more recent patient population in our study. Moreover, the median follow up in our study was much longer (49.5 vs. 20.5 months), which may be relevant regarding long-term survival results.

There is no doubt that IC remains more toxic than HMAs in older AML patients as reflected by the higher early death rate in our study. However, the rate of early death following induction chemotherapy has decreased over time, and new intensive chemotherapy formulations such as CPX-351 may further limit this risk and allow more patients to reach response and go to consolidation and/or maintenance therapy with novel agents such as CC-486 [32–34]. The age limit for allogeneic hematopoietic stem cell transplantation is also rising constantly and patients ≥ 70 years achieving complete remission may now become candidates for transplantation more and more frequently [35].

Obviously, these results should be discussed in light of recent advances in the treatment of AML patients judged unfit for IC. The addition of venetoclax to low-intensity therapies including HMA or LDAC has demonstrated significant efficacy by improving response rates and overall survival compared to HMA or LDAC single agents [10, 11]. In the VIALE-A study comparing venetoclax/azacitidine vs. placebo/azacitidine, the venetoclax experimental arm (median age, 76 years, de novo AML 75%, intermediate-risk cytogenetics 64%) induced a CR/CRi rate of 66.4%, which represents a real breakthrough in the field of low-intensity therapies, reaching the level of IC results. It is noteworthy that age ≥ 75 years or a performance status of 2 were isolated criteria sufficient to be judged ineligible for IC, which is debatable. Median overall survival was 14.7 months and two-year survival ~35%. Since previous studies have shown similar median overall survival between HMAs and IC as discussed above, it is tempting to speculate that venetoclax plus low-intensity therapies will therefore be superior to IC (and likely less toxic) in older AML patients. However, the median follow-up of VIALE-A (20.5 months) is not yet long enough to determine the long-term survival rate with this novel therapeutic approach. Moreover, the first real-world experience with venetoclax/azacitidine suggested an inferior outcome to the clinical trial results [36]. Therefore, prospective randomized trials will be necessary to answer this important clinical issue.

The main weakness of our study is its retrospective nature and the lack of predefined criteria to justify the therapeutic options proposed in real life. Molecular documentation remains insufficient, even if we could show the prognostic impact of *NPM1* and *FLT3*-ITD mutations. However, the multicenter and multinational aspect, as well as the very high number of patients allowed robust multivariate and matching analyses that shed light on the respective place of IC and HMAs in patients 70 years of age or older.

Although IC results remains largely unsatisfactory in AML of the elderly, it is associated with short-term toxicities but long-term survival in a sizeable number of patients. The main result of our study suggests that the evaluation of new alternative treatments should integrate long term survival as a relevant clinical endpoint in order to have a clear vision of the benefits of prolonged low-intensity treatments compared to short IC, even in elderly subjects.

## Supplementary information

Supplementary Figure 1

Supplementary Table 1

Supplementary Table 2

Supplementary Table 3

Supplementary Table 4

Supplementary Table 5
