# Supplementary material for: Long-term survival after intensive chemotherapy or hypomethylating agents in AML patients aged 70 years and older: a large patient data set study from European registries
Source: Leukemia. 2021 Nov 13;36(4):913–22. doi: 10.1038/s41375-021-01425-9 (PMC8979811; doi:10.1038/s41375-021-01425-9)

**Supplementary Figure 1**

**Legend : A.** Royston and Parmar adjusted* hazard ratio for overall survival in HMA vs. IC for each month from diagnosis – in patients < 75y (*Adjusted for performance status > 1, white blood cell count at diagnosis > 30 giga per liter, cytogenetic risk and secondary vs de novo AML). **B.** Royston and Parmar adjusted* hazard ratio for overall survival in HMA vs. IC for each month from diagnosis – in patients ≥ 75y (*Adjusted for performance status > 1, white blood cell count at diagnosis > 30 giga per liter, cytogenetic risk and secondary vs de novo AML). **C.** Royston and Parmar adjusted* hazard ratio for relapse-free survival in HMAs vs. IC for each month from CR/CRi – in patients < 75y (*Adjusted for performance status > 1, white blood cell count at diagnosis > 30 giga per liter, cytogenetic risk, secondary vs. de novo AML, *NPM1* and *FLT3*-ITD mutations). **D.** Royston and Parmar adjusted* hazard ratio for relapse-free survival in HMAs vs. IC for each month from CR/CRi – in patients ≥ 75y (*Adjusted for performance status > 1, white blood cell count at diagnosis > 30 giga per liter, cytogenetic risk, secondary vs. de novo AML, *NPM1* and *FLT3*-ITD mutation

**A**

**B**

**C**

**D**


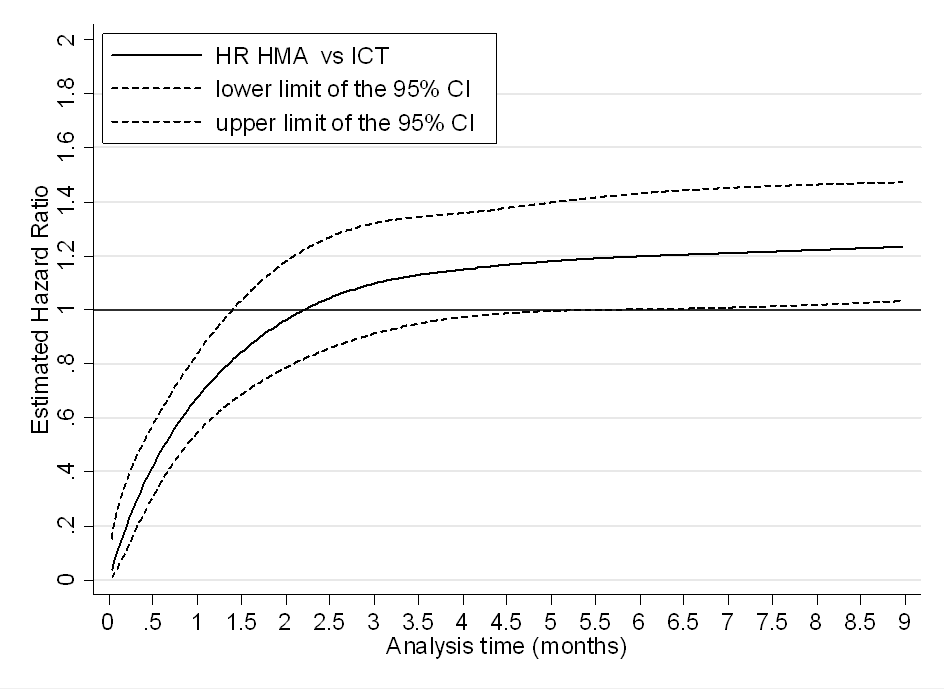

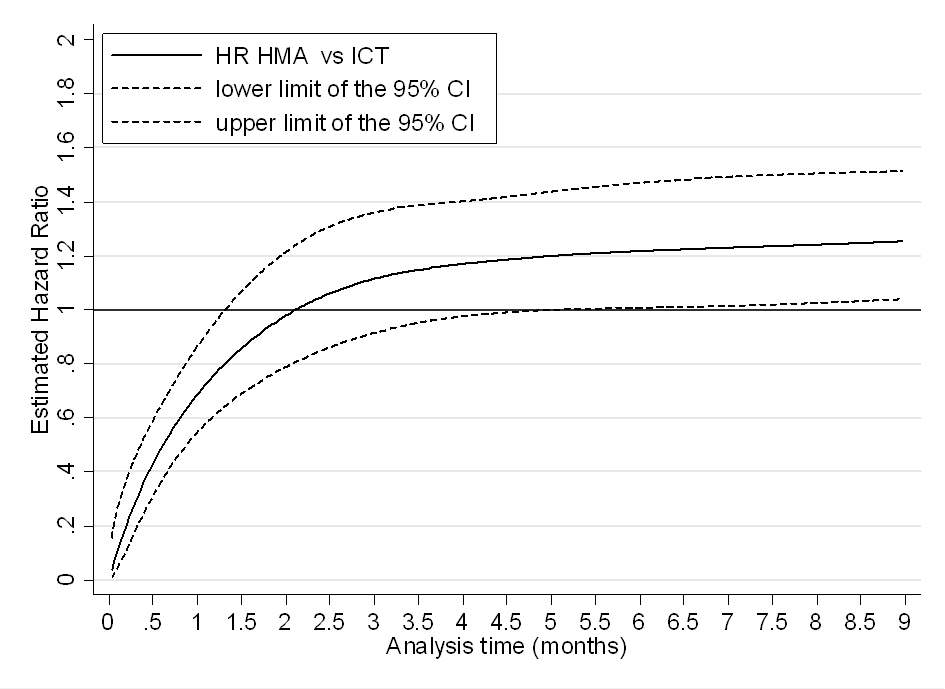

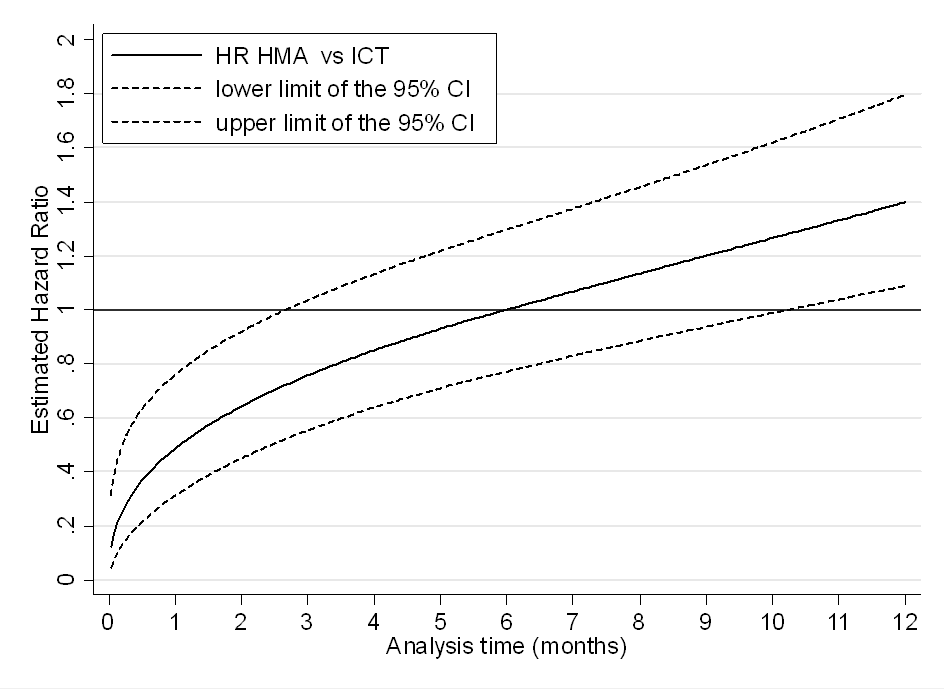

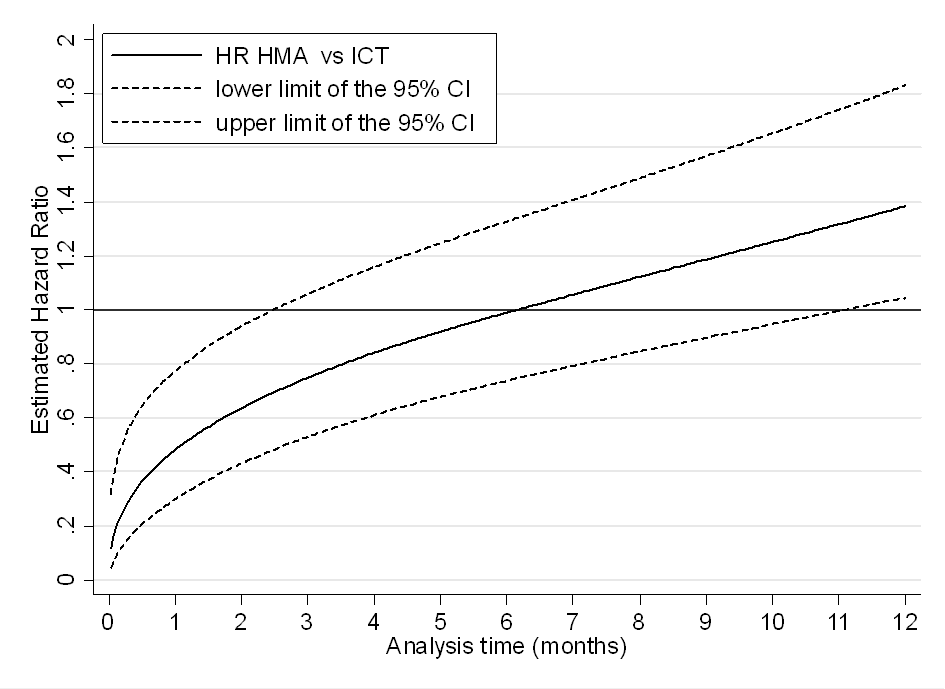

Supplement: Supplementary file 1 — Supplementary Figure 1 [file 41375_2021_1425_MOESM1_ESM.docx]
