# Supplementary material for: Long-term survival after intensive chemotherapy or hypomethylating agents in AML patients aged 70 years and older: a large patient data set study from European registries
Source: Leukemia. 2021 Nov 13;36(4):913–22. doi: 10.1038/s41375-021-01425-9 (PMC8979811; doi:10.1038/s41375-021-01425-9)
Supplement: Supplementary file 2 — Supplementary Table 1 [file 41375_2021_1425_MOESM2_ESM.docx]

**Supplementary Table 1: Characteristics of the 1 428 AML patients ≥ 70 years not selected for intensive chemotherapy or hypomethylating agents**

|  | **Semi-intensive chemotherapy**  **N=464 (32.5%)** | **Low-dose cytarabine**  **N=127 (8.9%)** | **Supportive care***  **N=837 (58.6%)** |
| --- | --- | --- | --- |
| **Study period – no. (%)**  2007-2012  2013-2018 | 127 (27.4)  337 (72.6) | 58 (45.7)  69 (54.3) | 319 (38.1)  518 (61.9) |
| **Sex – no. (%)**  Male  Female | 265 (57.1)  199 (42.9) | 71 (55.9)  56 (44.1) | 468 (55.9)  369 (44.1) |
| **Age – years**  Median (IQR)  < 75y – no. (%)  ≥ 75y – no. (%) | 76.8 (73.5-80.2)  168 (36.2)  296 (63.8) | 78.0 (75.9-81.9)  21 (16.5)  106 (83.5) | 80.2 (76.3-85.0)  147 (17.6)  690 (82.4) |
| **ECOG performance status – no. (%)**  0-1  2-4 | 304 (69.2)  135 (30.8) | 60 (55.0)  49 (45.0) | 233 (38.4)  373 (61.6) |
| **AML status – no. (%)**  De novo  Secondary | 280 (64.8)  152 (35.2) | 53 (45.3)  64 (54.7) | 407 (55.0)  333 (45.0) |
| **White blood cell count – giga per liter**  Median (IQR)  ≤ 30 – no. (%)  > 30 – no. (%) | 7.7 (2.5-34.0)  326 (73.6)  117 (26.4) | 12.9 (2.5-56.3)  81 (64.3)  45 (35.7) | 8.8 (2.3-38.0)  542 (70.6)  226 (29.4) |
| **Peripheral blasts – %**  Median (IQR) | 22.0 (7.0-60.0) | 20.5 (4.0-62.0) | 18.0 (4.0-49.0) |
| **Bone marrow blasts - %**  Median (IQR) | 55.0 (32.0-76.0) | 54.0 (32.0-80.0) | 43.5 (29.0-70.0) |
| **LDH – IU/liter**  Median (IQR) | 444.0 (274.0-709.0) | 478.5 (295.0-1028.0) | 486.0 (299.0-854.0) |
| **Cytogenetic risk – no. (%)**  Favorable  Intermediate  Adverse | 10 (2.8)  250 (70.4)  95 (26.8) | 0 (0.0)  75 (72.8)  28 (27.2) | 7 (1.2)  344 (57.0)  253 (41.9) |
| ***NPM1* mutations – no. (%)**No  Yes | 171 (72.8)  64 (27.2) | 65 (80.2)  16 (19.8) | 204 (74.5)  70 (25.5) |
| ***FLT3*-*ITD* mutations – no. (%)**  No  Yes  Allelic ratio – no  Median (IQR) | 207 (83.5)  41 (16.5)  0.7  (0.5-0.8) | 61 (80.3)  15 (19.7)  0.6  (0.1-0.7) | 225 (83.6)  44 (16.4)  0.5  (0.3-0.8) |
| ***IDH1*-*R132* mutations – no. (%)**  No  Yes | 70 (94.6)  4 (5.4) | 13 (86.7)  2 (13.3) | 123 (93.9)  8 (6.1) |
| ***IDH2-R140* mutations – no. (%)**  No  Yes | 67 (94.4)  4 (5.6) | 14 (93.3)  1 (6.7) | 122 (91.7)  11 (8.3) |
| ***IDH2-R172* mutations – no. (%)**  No  Yes | 69 (97.2)  2 (2.8) | 15 (100.0)  0 (0.0) | 130 (98.5)  2 (1.5) |
| **Inclusion in a clinical trial – no. (%)**  No  Yes | 378 (81.5)  86 (18.5) | 103 (81.1)  24 (18.9) | 832 (99.4)  5 (0.6) |
| **Allogeneic stem cell transplantation – no. (%)**  No  Yes | 464 (100.0)  0 (0.0) | 127 (100.0)  0 (0.0) | 837 (100.0)  0 (0.0) |

* Included in DATAML and SAL registries
