# Supplementary material for: Long-term survival after intensive chemotherapy or hypomethylating agents in AML patients aged 70 years and older: a large patient data set study from European registries
Source: Leukemia. 2021 Nov 13;36(4):913–22. doi: 10.1038/s41375-021-01425-9 (PMC8979811; doi:10.1038/s41375-021-01425-9)
Supplement: Supplementary file 3 — Supplementary Table 2 [file 41375_2021_1425_MOESM3_ESM.docx]

**Supplementary Table 2: Outcome of the 1 428 AML patients ≥ 70 years not selected for intensive chemotherapy or hypomethylating agents**

|  | **Semi-intensive chemotherapy**  **N=464 (32.5%)** | **Low-dose cytarabine**  **N=127 (8.9%)** | **Supportive care***  **N=837 (58.6%)** |
| --- | --- | --- | --- |
| **Median follow-up - months (95%CI)** | 36.1 (20.2-55.2) | 37.5 (19.8-37.5) | 40.7 (6.2-79.5) |
| **CR/CRi – no. (%)** | 145 (31.2) | 14 (11.0) | NA |
| **Day-30 death – no. (%)**  **Day-60 death – no. (%)** | 74 (15.9)  125 (26.9) | 32 (25.2)  47 (37.0) | 282 (33.7)  420 (50.2) |
| **Median overall survival - months (95%CI)** | 4.80 (4.17-5.82) | 3.61 (2.60-5.10) | 1.5 (1.31-1.68) |
| **Overall survival - % (95%CI)**  1 year  3 year  5 year | 27.1 (23.0-31.4)  6.4 (4.1-9.3)  2.0 (0.6-5.3) | 22.3 (15.1-30.3)  1.2 (0.1-5.6)  - | 6.4 (4.7-8.5)  1.1 (0.5-2.3)  0.4 (0.05-1.6) |

* Included in DATAML and SAL registries; NA: Not applicable
