# Supplementary material for: Long-term survival after intensive chemotherapy or hypomethylating agents in AML patients aged 70 years and older: a large patient data set study from European registries
Source: Leukemia. 2021 Nov 13;36(4):913–22. doi: 10.1038/s41375-021-01425-9 (PMC8979811; doi:10.1038/s41375-021-01425-9)
Supplement: Supplementary file 4 — Supplementary Table 3 [file 41375_2021_1425_MOESM4_ESM.docx]

**Supplementary Table 3: Association between CR achievement and long term overall survival**

**CR in long-term survival (>3 years) patients**

|  | **CR** | |  |  |
| --- | --- | --- | --- | --- |
|  | **No** | **Yes** | **p value** | **Total** |
|  | N=40 (18.9%) | N=172 (81.1%) |  | N=212 (100%) |
| 1L treatment n(%) |  |  | <.0001* |  |
| IC | 10 (6.2) | 151 (93.8) |  | 161 (75.9) |
| HMA | 30 (58.8) | 21 (41.2) |  | 51 (24.1) |
|  |  |  |  |  |

*OR=0.05 [95%CI : 0.02-0.11] for CR in HMA versus IC group*

**CR in no long-term survival patients**

|  | **CR** | |  |  |
| --- | --- | --- | --- | --- |
|  | **No** | **Yes** | **p value** | **Total** |
|  | N=1348 (65.4%) | N=712 (34.6%) |  | N=2060(100%) |
| 1L treatment n(%) |  |  | <.0001* |  |
| IC | 516 (49.7) | 522 (50.3) |  | 1038 (50.4) |
| HMA | 832 (81.4) | 190 (18.6) |  | 1022 (49.6) |
|  |  |  |  |  |

*OR=0.23 [95%CI : 0.18-0.28] for CR in HMA versus IC group*
