# Supplementary material for: Long-term survival after intensive chemotherapy or hypomethylating agents in AML patients aged 70 years and older: a large patient data set study from European registries
Source: Leukemia. 2021 Nov 13;36(4):913–22. doi: 10.1038/s41375-021-01425-9 (PMC8979811; doi:10.1038/s41375-021-01425-9)
Supplement: Supplementary file 5 — Supplementary Table 4 [file 41375_2021_1425_MOESM5_ESM.docx]

**Supplementary Table 4: Complete remission according to age (70-74 vs ≥ 75years)**

|  | **Number** | **Events** | **aOR** | **95% CI** | ***P*-value** |
| --- | --- | --- | --- | --- | --- |
| **Age < 75years** | **CR/CRi** | | | | |
| **Treatment**  Intensive chemotherapy  Hypomethylating agents | 740  331 | 449  76 | 1  0.24 | 0.18-0.33 | <0.001 |
| **Age ≥ 75years** | **CR/CRi** | | | | |
| **Treatment**  Intensive chemotherapy  Hypomethylating agents | 459  742 | 224  135 | 1  0.26 | 0.19-0.34 | <0.001 |

aOR, adjusted odds ratio (adjustment for age ≥ 75y, performance status > 1, white blood cell count at diagnosis > 30 giga per liter, cytogenetic risk, secondary vs de novo AML and NPM1 mutation); CI, confidence interval; CR, complete remission, CRi, complete remission with incomplete hematologic recovery.
