# Supplementary material for: Long-term survival after intensive chemotherapy or hypomethylating agents in AML patients aged 70 years and older: a large patient data set study from European registries
Source: Leukemia. 2021 Nov 13;36(4):913–22. doi: 10.1038/s41375-021-01425-9 (PMC8979811; doi:10.1038/s41375-021-01425-9)
Supplement: Supplementary file 6 — Supplementary Table 5 [file 41375_2021_1425_MOESM6_ESM.docx]

**Supplementary Table 5: Pairwise comparison between intensive chemotherapy and hypomethylating agents using propensity score matching**

|  | **Number** | **Events** | **OR** | **95% CI** | ***P*-value** |
| --- | --- | --- | --- | --- | --- |
|  | **CR/CRi** | | | | |
| **Treatment**  Intensive chemotherapy  Hypomethylating agents | 532  532 | 279  112 | 1  0.24 | 0.18-0.32 | <0.001 |
|  | **Day-30 death** | | | | |
| **Treatment**  Intensive chemotherapy  Hypomethylating agents | 532  532 | 72  41 | 1  0.53 | 0.36-0.80 | 0.002 |
|  | **Day-60 death** | | | | |
| **Treatment**  Intensive chemotherapy  Hypomethylating agents | 532  532 | 116  89 | 1  0.72 | 0.53-0.98 | 0.036 |

OR, odds ratio; CI, confidence interval.
